# Advancing sustainable beef through research and collaboration

**DOI:** 10.1093/af/vfag045

**Published:** 2026-10-19

**Authors:** Matthew Beck, Jude Capper, Kristen Johnson

**Affiliations:** Department of Animal Science, Texas A&M University, College Station, TX 77843; Department of Agriculture and Environment, Harper Adams University, Newport, TF10 8NB, UK; Department of Animal Science, Washington State University, Pullman, WA 99164

**Keywords:** greenhouse gas emissions, carbon footprint, water quality, ecosystem health, human nutrition

Implications

- Sustainable food systems are challenging, as they encompass environmental, economic, and social dynamics, which require subjective discernment based on individual values. As such, what is considered sustainable by one individual may not be deemed sustainable for another.
- The beef industry has made significant strides towards becoming a sustainable food system; however, as with all industries, there is room for improvement. In this case, the sustainability domains that have the largest potential for improvement are air, water, and land.
- However, research undertaken to enhance beef industry sustainability must be transdisciplinary and collaborative to ensure that strategies do not cause unintended consequences or pollution swapping.
- Recognizing the complexity of the task ahead of the beef industry, a collaborative roadmapping workshop was conducted to bring together researchers with a range of expertise to identify knowledge gaps. This workshop aimed to develop a strategic approach to future research in enhancing beef sustainability, and this special issue of Animal Frontiers stems from the workshop.

## Introduction

Sustainability can be defined as meeting our needs today without compromising our capabilities to meet our needs in the future (Brundtland, 1987). Classically, sustainability encompasses environmental, economic, and social pillars and the interactions and trade-offs therein. If a single aspect of sustainability is focused upon, trade-offs may result in other metrics, leading sustainability to be considered as a “wicked problem,” where no right or wrong solution exists, only better or worse (Rittel and Webber, 1973). Furthermore, as the three pillars of sustainability are multifaceted, measured on different scales (dollars, kg of carbon dioxide equivalent (CO_2_e), water quality, ecosystem health, etc.), and vary in importance according to individual values, classifying a production system as sustainable or not is eminently subjective (Rowley et al., 2012). Given this subjectivity, a system or practice that one stakeholder might consider sustainable could be considered unsustainable by another. The U.S. beef industry provides a case study for the global industry and provides an example of this dichotomy.

Manzano et al. (2023) suggested that the global beef industry is already sustainable through providing economic gains, food security, landscape maintenance, and multiple other benefits. However, as with every industry, there is room for improvement—in this case by focusing on air, water, and land issues. Research ­progress and strategies to improve these domains must take a ­collaborative, holistic approach to avoid unintended trade-offs or pollution swapping, where improving one sustainability metric hinders another. No single researcher has all the requisite knowledge to embrace all three domains, and transdisciplinary collaboration is required across multiple areas of expertise to understand and develop management practices that will enhance beef production sustainability. Realizing this, the U.S. Roundtable for Sustainable Beef (USRSB) hosted a roadmapping workshop in October 2024. This workshop convened 80 experts from 50 different organizations to identify knowledge gaps and therefore facilitate opportunities for future collaborative research (USRSB, 2025). The current ­special issue is a result of this original workshop and aims to coalesce the findings. The remainder of this introduction provides a brief overview of the manuscripts that make up this special issue.

## The Need for an Action Plan

The beef industry is unique in being segregated across cow-calf, stocker/backgrounder, and finisher sectors. Each sector comprises a wide range of stakeholders varying in geographic location, climatic conditions, operation size, resource access, and priorities. To develop and implement sustainable improvements in air, water, and land domains, strategies must be region-specific, with minimal unintended consequences. Consequently, the need exists for a coordinated plan, with transdisciplinary and cross-sector collaboration. Lai et al. (2026) describes and discusses this need, further extrapolating on the need for an action plan (i.e., a research roadmap). Importantly, although the U.S. beef industry is already one of the most efficient in the world, they highlight that prioritizing research to enhance U.S. beef industry sustainability will also extend solutions globally.

## The Roadmap

Scientists within the USRSB workshop determined that air and greenhouse gas emissions (GHGe), water, and land were the domains within the U.S. beef industry with the greatest opportunities for sustainability improvement. Subject area expert teams leading these discussions have authored publications related to these domains within the current special issue and are described in the remainder of this section.

Enteric methane emissions represent the largest contributor to GHGe from the beef industry, and the majority of total GHGe arise from the pastoral cow-calf sector. Ammonia and volatile organic compound emissions also arise from beef production, and while these emissions are not necessarily greenhouse gases, they can damage vulnerable ecosystems and lead to poor air ­quality. Thompson et al. (2026) broadly describe where the beef industry currently stands in terms of gas emissions, the knowledge that needs to be generated, and areas in which potential mitigation strategies exist. A major conclusion from this research is that there is no single “silver bullet” solution to this challenge, but that advances must be made across multiple research fields.

Stanley et al. (2026) discuss the status, gaps, and challenges facing grazing land science across four critical research areas: grazing management, soil carbon, other ecological indicators, and land use. The authors describe the general underlying ­principles of grazing management and the potential wider adoption of adaptive grazing management. Some of the challenges identified include inadequate record keeping, inconsistent terminology in peer-reviewed literature, difficulties in conducting long-term grazing management studies, and accounting for ­spatial soil carbon variability.

Water availability is a significant driver of regional cattle production practices, and water quality must be maintained to avoid ecological issues and to protect potability in both grazing and feedlot systems. Wagner et al. (2026) describe important issues facing these enterprises and describe steps that should be taken to increase beef system resilience. In grazing systems, water ­quality, drought severity, and low adoption of best management ­practices reduce system sustainability. The authors identify management practices to protect riparian areas, adaptive grazing management, precision livestock technologies, and coordination between Extension and USDA-NRCS personnel as important solutions to these issues. Water-related challenges to feedlots identified by the authors include nutrient runoff, high water use for feed production, and diminishing water availability. Manure management, recycling wastewater, and development of low-water forages for feed production were identified as promising strategies for feedlot production. Data requirements at the water-shed scale, greater technology testing and validation, practical water reclamation strategy development, and drought-tolerant crops are important research areas going forward (Wagner et al. 2026).

## From Roadmap to Action

Research and innovation in the U.S. beef supply chain has led to substantial improvements in efficiency and reduction in environmental impacts on a per unit of product basis. Werth et al. (2026a) describes several of these innovations, including emerging technologies across the air, land, and water domains. These authors proceeded to outline six research priorities to enhance the sustainability of beef production. These include development of grazing management plans, optimization in the feedyard, technology innovation, model development to evaluate impacts, genetic improvement, and producer practicality and trade-offs to implement strategies (Werth et al. 2026a).

Planning a sustainable beef research roadmap will ultimately be unsuccessful unless action occurs and deliverables are met. Werth et al. (2026b) describes three main phases toward coordinated research and action. These phases span short (0–2 years), medium (2–5 years), and long-term (5+ years) horizons and represent a programmatic approach, where a framework is established with aligned sustainability indicators. A key aspect of the needed framework is a secure data sharing platform and relevant program governance. Regional expansion then leads to the integration of producer-led research trials. Finally, deployment of decision support tools to aid producers and other stakeholders is needed to enhance system resilience and sustainability. To achieve these goals, there must be access to funding, and Werth et al. (2026b) discusses a mechanism to raise these funds. The following section will describe the manuscripts that further describe funding mechanisms but also other challenges and opportunities facing the beef industry to enhance its sustainability.

### Addressing challenges to implementation

Reducing GHGe offers a significant opportunity to enhance global beef industry sustainability, yet accounting for biogenic methane, the predominant GHGe produced by beef production, has a key influence on this metric. Typically, GHGe are expressed as CO_2_e based on the global warming potential (GWP) on a 100-year time horizon (GWP100). However, methane only lasts in the atmosphere for approximately 12 years. Consequently, the warming impacts of methane are highly dependent on changes in emission rates over time. This nuance is not adequately captured by GWP100, and, as such, targets based on this metric may not align with actual temperature responses. Jones et al. (2026) recommend a split-gas accounting framework so that proposed targets can more appropriately align with desired outcomes. The authors provide examples of how this change in accounting resulted in New Zealand updating their biogenic methane targets to a more achievable goal, which was still aligned with their intended outcome.

The sum of management outcomes within a given region impacts the ecological functions of landscapes—whether positive or negative. To enhance the ecosystem function of the wider landscape, management strategies with nature-positive outcomes must be adopted by multiple cattle producers within that region. Crouch et al. (2026) describe governance and multi-stakeholder platforms for establishing landscape and jurisdictional approaches to achieve this goal. This manuscript outlines the problems facing landscape-level regions, including ecosystem function, leakage and rebound risk, and barriers due to traceability, appropriate metrics, and baselines, concluding by outlining how governance frameworks can be implemented.

Circularity occurs where resources are used for as long as possible through reuse, recycling, and regeneration of materials or products with important environmental implications (Werth et al., 2026c). Beef cattle often consume byproduct feeds, which would otherwise be wasted, and produce nutrient-rich fertilizers in the form of manure. As such, the U.S. beef industry already fills an important role in food production circularity, although its contribution is poorly understood. Werth et al. (2026c) outlines this circularity niche, describes how beef production aligns with the five principles of circularity, and outlines opportunities to expand its circularity, enhancing beef industry sustainability.

Cleveland et al. (2026) describes how genetics represents an important strategy for increasing efficiency, reducing environmental impacts of beef production, and can advance system sustainability. Through genetic selection, producers can cumulatively enhance their cattle’s productivity, feed efficiency, and profitability, all while reducing environmental impact. This is a win-win opportunity for producers to reduce GHGe while improving economic viability. However, Cleveland et al. (2026) also identified bottlenecks and important guardrails that are needed. Firstly, genetic selection is largely limited by our capacity to collect phenotypic trait data. Secondly, directly selecting for lower enteric methane must be economically incentivized before producers adopt it. Finally, genetic selection should not be at the expense of locally adapted populations which are resilient to specific environments.

Beef is a source of high-quality protein and bioavailable micronutrients, which are often deficient in human diets (Sekula et al., 2026). Dietary guidelines making recommendations based on frameworks that weigh nutritional, health, and environmental aspects have recently become popular, yet the relative weightings of these factors can result in considerably different recommendations for red meat consumption. Sekula et al. (2026) recommend a holistic viewpoint when developing dietary guidelines that balance nutritional and sustainability considerations.

Animal welfare is an important ethical consideration for livestock production and is a prerequisite for efficient and profitable cattle production. If cattle have poor welfare and health outcomes, they will be unproductive and less profitable. Warboys and Petre (2026) discuss how cattle welfare, health, and husbandry practices interact with sustainability. There are clear economic incentives for producers to provide their livestock with appropriate welfare and to adopt best husbandry practices while raising their cattle. This manuscript also highlights the environmental benefits that these practices induce and highlights that low- and middle-income countries may have the most to gain from changes in farm practice.

Any transdisciplinary research program requires funding, and producers will often require access to additional funding resources to make operational changes, even if those practices would increase their profitability in the long term. Access to funding represents a considerable roadblock to enhancing global beef industry sustainability. Talbott et al. (2026) discuss several financial mechanisms and investments to drive sustainable change, using specific global case studies demonstrating how these mechanisms have previously been employed.

## Conclusions

The U.S. beef industry may already be considered sustainable given its multifactorial roles in byproduct feed use, landscape maintenance, provision of food security, and economic gains. Nevertheless, there is room for improvement, particularly within the air, water, and land domains. The complexity and segregation within the industry, in addition to the variety of stakeholders with differing priorities, establishes the need for establishing a transdisciplinary and cross-sector research collaboration that provides tools to enhance beef industry sustainability. Such a collaboration would develop practices that are universally applicable while remaining regionally adaptable and that would ensure minimal unintended consequences. Ultimately, beef producers require tools that increase the profitability of their operation while reducing their environmental impact across air, water, and land domains.

*Conflict of interest statement*. M. Beck, J. Capper, and K. Johnson served as guest editors for this issue of *Animal Frontiers*. They played no role in the review or decisions regarding this manuscript. The authors declare no other real or perceived conflicts of interest.

## Author Biographies

**Matthew Beck** graduated with his B.S. (2015) and M.S. (2017) in Animal Science from Oklahoma State University. He then completed his Ph.D. in Animal Science at Lincoln University in Lincoln, New Zealand, in 2020. Following his schooling, Matt served as a Research Animal Scientist with the USDA-ARS in Bushland, Texas, for three years. He then started his current role as Assistant Professor of Beef Cattle Nutrition within the Department of Animal Science of Texas A&M University in October of 2024. Throughout his career, Matt’s research has focused on enhancing the sustainability of beef production from increasing system productivity and profitability to reducing environmental impacts.

**Jude Capper** is the Chair and Professor of Sustainable Ruminant Production at Harper Adams University in the UK and is also an independent Livestock Sustainability Consultant. Jude’s research focuses on the sustainability of dairy, beef, and sheep production systems, and she is currently working on research projects relating to on-farm greenhouse gas emissions from ruminant production and the impacts of livestock health on sustainability. Jude is Vice-Chair of the National Beef Association and a liveryman of the Worshipful Company of Butchers. She currently chairs the Red Tractor Beef & Lamb Technical Advisory Committee, responsible for UK farm assurance standards within that sector.

**Kristen Johnson** is a Professor Emeritus at Washington State University. Her research focuses on the sustainability of beef and dairy production systems. This includes greenhouse gas and ammonia measurements to examine mitigation strategies and provide the livestock industries with information with which decisions can be made. Current projects include developing case studies for dairy producers to evaluate the economics of manure management changes and characterization of byproduct use by feedlot operators.
